# Action Monitoring Through External or Internal Focus of Attention Does Not Impair Endurance Performance

**DOI:** 10.3389/fpsyg.2019.00535

**Published:** 2019-03-13

**Authors:** Francesca Vitali, Cantor Tarperi, Jacopo Cristini, Andrea Rinaldi, Arnaldo Zelli, Fabio Lucidi, Federico Schena, Laura Bortoli, Claudio Robazza

**Affiliations:** ^1^Department of Neurosciences, Biomedicine and Movement Sciences, University of Verona, Verona, Italy; ^2^Department of Movement, Human and Health Sciences, Foro Italico University of Rome, Rome, Italy; ^3^Department of Developmental and Social Psychology, La Sapienza University of Rome, Rome, Italy; ^4^BIND–Behavioral Imaging and Neural Dynamics Center, Department of Medicine and Aging Sciences, Università degli Studi G. d’Annunzio Chieti e Pescara, Chieti, Italy

**Keywords:** action monitoring, attentional focus, fatigue, hedonic tone, multi-action plan model, endurance

## Abstract

Attentional focus in endurance sports has been found to largely affect performance. To deal with discomfort, fatigue, and pain associated with endurance performance under pressure, athletes tend to direct attention to both internal (e.g., bodily) sensations and external (e.g., environmental) stimuli. The purpose of this study, framed within the multi-action plan (MAP) model, was to examine whether different levels of action monitoring through external or internal focus of attention could influence endurance performance. Action monitoring has been conceptualized as awareness of the current experience without necessarily influencing the course of action or disrupting automated motor processes. Thirty-two male participants (*M*_age_ = 29.12 years, *SD* = 6.12 years) were engaged in a treadmill, time-to-exhaustion running task across seven visits to the laboratory (i.e., task familiarization, baseline, four experimental conditions, and follow up). Assessment involved performance (i.e., time to exhaustion), oxygen uptake (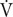
O_2_), blood lactate levels, ratings of perceived exertion (RPE), and perceived arousal and hedonic tone. Across four visits, participants were prompted to use the four attentional strategies (one per session) deriving from the interaction of low/high conscious monitoring level by external/internal attention focus in a counterbalanced experimental design. Repeated measures analysis of variance did not yield significant results in any variable of the study, performance included. Consistent with predictions of the MAP model, study findings showed that participants were able to attain same performance levels irrespective of whether they used a high or low level of action monitoring through an external or internal focus of attention. Findings suggest practical indications to help athletes deal with stress in endurance sports.

## Introduction

Attentional focus in endurance sports, such as running, cycling, orienteering, swimming, triathlon, rowing, and cross-country skiing, has been acknowledged to largely impact performance (e.g., Brick et al., 2016a; McCormick et al., 2018; Robazza et al., 2018). To deal with task demands and perform optimally, athletes need to pay attention to and monitor both internal (e.g., bodily) sensations and external (e.g., environmental) stimuli, especially under competitive pressure (Carson and Collins, 2016; Gropel, 2016; Buchanan et al., 2018). Over the last decades, cognitive functioning and motivational issues in endurance sports have received increased research interest, and study findings have been interpreted in light of different theoretical approaches in the attempt to understand decision making and metacognitive processes (e.g., Smits et al., 2014; Brick et al., 2016b), attentional focus and cognitive control (e.g., Brick et al., 2014, 2016a), and mental fatigue (e.g., Marcora et al., 2009; Marcora and Staiano, 2010; Boccia et al., 2018). Beyond psychological approaches, the role of central fatigue in endurance tasks has been explained in light of metabolic, neurochemical, physiological, and psychophysiological processes (e.g., Martin et al., 2018; McMorris et al., 2018; Pedrinolla et al., 2018). Notwithstanding the substantial progresses in the field, and general agreement of the importance of self-monitoring processes to deal with physical and mental exertion, a number of questions remain to be investigated. In this regard, a specific research question that we addressed in the current study is whether different levels of action monitoring through external or internal focus of attention could influence endurance performance.

In one of the most influential works on marathon runners, Morgan and Pollock (1977) differentiated between an associative (task related) and a dissociative (task unrelated) attentional strategy. This classification was based on the observation that elite runners tended to monitor (i.e., to associate) sensory information and adjust their pace accordingly, whereas less expert runners tended to focus more on distracting stimuli (i.e., to dissociate) to divert attention away from physical strain, exhaustion, pain, and mental exertion. Later, Stevinson and Biddle (1998, 1999) proposed a two-dimensional classification system by adding an internal-external (body related/unrelated) dimension to the associative-dissociative (task related/unrelated) dimension. In a muscular endurance task (i.e., a wall-sit isometric posture), Lohse and Sherwood (2011) combined the internal-external with the associative-dissociative dimensions. In particular, the wall-sit task was completed under three attention focus conditions: internal-associative (thighs position), external-associative (drawing imaginary lines between knee and hip), and external-dissociative (drawing imaginary lines between pylons in front of the participant). While the two types of external focus were equally effective, they were superior to the internal focus in increasing the time taken to failure and in reducing perceived exertion. More recently, Brick et al. (2014) proposed a working model to better categorize cognitive processes. They suggested an extension to the internal associative category of Stevinson and Biddle (1998) classification to include internal sensory monitoring (e.g., breathing, muscles soreness, and fatigue) and active self-regulation (e.g., cadence, pacing, technique, strategy, maintaining a relaxed state). They also differentiated between active, voluntary distraction and involuntary distraction (passive thoughts).

Despite the large body of research in endurance sports, (Schücker et al., 2016a,b) noted that research findings regarding attentional focus effects on endurance performance are controversial. An external focus of attention on the intended movement effects has been found to benefit movement efficiency in endurance activities (e.g., optimized muscular activity and oxygen consumption), as well as motor learning, movement effectiveness (e.g., precision in hitting a target, exerting a specific amount of force, keeping balance) independent of skill level, task, and age (Wulf, 2007, 2013). This effect has been explained with the constrained action hypothesis (Wulf et al., 2001). According to this hypothesis, an external attentional focus determines a more automatic mode of control through unconscious and fast processes of movement control. In contrast, an internal focus on movement execution is contended to induce a conscious type of control that interferes with automatic control processes regulating movement coordination (Wulf, 2007).

Although performance advantages of an external focus of attention have been found across many studies conducted on discrete and short-time motor tasks (for reviews, see Wulf, 2013; Wulf and Lewthwaite, 2016), controversial research findings have been reported in endurance sports and long-lasting cyclic motor tasks requiring sustained attention over time (see Brick et al., 2014). One explanation of the inconsistent results can lie on the equivocal or vague definition of the notion of attention focus operationalized in different manners. For example, an internal focus of attention can involve physical sensations or technique, while an external focus can include visual or auditory information. It is therefore unsurprising that studies manipulating attention in endurance sports led to different practical recommendations on whether it is more beneficial to focus attention internally or externally. Schücker et al. (2014) tried to resolve this issue by classifying an internal focus of attention into two subclasses: A focus on physical sensations exerting beneficial effects on performance, and a focus on automated processes exerting detrimental effects. Schücker et al. (2014) used this framework in a study on runners involved in a 24 min treadmill task. In particular, they examined the effects on running economy of an internal focus on physical sensations and an internal focus on automated processes. In the internal focus on physical sensations, participants were asked to direct attention on feelings of the body, perceived effort, and body responses during the exercise. In the internal focus on automated processes runners were asked either to pay attention to their breathing dynamic or to monitor their running movement (i.e., feet and legs action). According to the study hypotheses, both the internal foci of attention directed on automated processes (i.e., breathing or running movement) were detrimental to movement economy measured through oxygen consumption (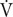
O_2_), whereas a focus on internal physical sensations did not impair movement efficiency.

The finding that an internal focus is not harmful as long as it does not disrupt automated processes is not in contrast with the constrained action hypothesis (Wulf et al., 2001) or other theoretical approaches, such as reinvestment theory (Masters, 1992; Masters and Maxwell, 2008) and explicit monitoring theory (Beilock and Carr, 2001; Beilock, 2011). All approaches, indeed, agree that focusing attention on movement execution impairs automaticity. As van Ginneken et al. (2017) pointed out, the beneficial or detrimental effects of an internal focus may depend on its relative emphasis on conscious monitoring or control, respectively. Monitoring involves movements “observation” and awareness of what is currently happening without necessarily influencing the course of action. This is much like a “mindful” moment-to-moment awareness and task relevant attention on current behavior. A mindful attitude is contended to promote a modified relationship with internal experiences (i.e., cognitions, emotions, and physiological sensations) rather than trying to change them (Gardner and Moore, 2007). Conversely, control entails both observation and influence—a conscious manipulation of the mechanics of the action during motor output (Masters and Maxwell, 2008).

van Ginneken et al. (2017) compared the effects of conscious monitoring and conscious control on performance in a darts task using specific instructions to manipulate both internal and external foci of attention. Conscious monitoring instructions emphasized awareness of arm movements (internal focus) or awareness of dart flight (external focus), whereas conscious control requests were to produce an ideal arm movement (internal focus) or an ideal dart flight (external focus). Based on the results of this study and previous research (e.g., Malhotra et al., 2015), the authors concluded that conscious monitoring can leave motor performance unaffected or even influence it positively (Zhang et al., 2016), whereas conscious control disrupts motor performance. Predictions concur with the reinvestment theory (Masters and Maxwell, 2008), explicit monitoring theory (Beilock and Carr, 2001), and the constrained action hypothesis (Wulf et al., 2001). Although using different perspectives, the three approaches place emphasis on the detrimental effects deriving from trying to consciously control the course of the action. However, the effect of consciously monitoring the action has not been examined or explicitly recognized in the different theoretical views.

The contention that an internal focus of attention is not always associated with detrimental effects and that can even benefit performance is also endorsed by action and motor-centered frameworks (Hanin and Hanina, 2009; Carson and Collins, 2016; Hanin et al., 2016; for a review, see Robazza and Ruiz, 2018). The multi-action plan (MAP) model, in particular, has been proposed to account for the multiple performance states of athletes, and to help them reach and maintain high execution standards in training and competition (Bortoli et al., 2012; Robazza et al., 2016). In the MAP model, high and low levels of performance are categorized in function of high and low levels of action control. In essence, four performance states are identified through this interaction: (1) Type 1, optimal-automated performance state, in which high performance is attained with a low focus of attention aimed at “supervising” the correct flow of movement execution; (2) Type 2, optimal-controlled performance, wherein high performance is reached through attention focused on the core component(s) of the action to prevent step-by-step movement control and to ensure the action be properly run; (3) Type 3, non-optimal-controlled performance, with undue and effortful attentional focus toward action control; and (4) Type 4, non-optimal-automated performance, with unfocused or wandering attention. In a laboratory cycling task (Bertollo et al., 2015; di Fronso et al., 2018), participants were asked to focus attention on a metronome reproducing an individual preferred pedaling rate (i.e., Type 1 state, external attention) or to pay attention to the preferred pedaling rate (i.e., Type 2 state, internal attention). Both conditions led participants to attain a better time-to-exhaustion performance than attending to feelings of muscle pain, tension, and fatigue (i.e., Type 3 state, internal attention). No differences were found in terms of performance outcomes between Type 1 and Type 2 states.

The Schücker et al. (2014) classification of attention focus either on physical sensations or on automated processes, as well as the van Ginneken et al. (2017) proposal to differentiate between monitoring and controlling the action, could be framed within the MAP conceptualization (Bortoli et al., 2012; Robazza et al., 2016). Specifically, directing attention to physical sensations in the running task (Schücker et al., 2014) can be viewed as a mental strategy to attain a Type 2 state functional for performance, whereas attempts to control automated processes would determine a Type 3 dysfunctional state. In the same vein, action monitoring and action control in darts throwing (van Ginneken et al., 2017) can be considered as characterizing a Type 1 functional state and a Type 3 dysfunctional state, respectively. A common assumption across the outlined perspectives is that a monitoring (“mindful,” “supervising,” and attending to action core components) attitude is beneficial, whereas controlling automated motor processes is detrimental. Although scant, research evidence is in favor of action monitoring rather than action control. What remains to be established is whether different levels of external or internal monitoring influence performance differently. Thus, the purpose of the current study was to determine whether low or high levels of external or internal conscious monitoring influence performance differently in a treadmill endurance task.

To manipulate the level of conscious monitoring, we alternated task requirements during execution according to the contextual interference paradigm applied to the learning of motor skills (for review, see Farrow and Buszard, 2017). Low contextual interference (low cognitive load) is created when different tasks are executed randomly or in serial order, one after another, usually allowing enough time to the performer to familiarize with one task before switching to the other. High contextual interference (high cognitive load) occurs when tasks change more frequently in random order or serial order, thus leaving less time to the performer to become acquainted. A high contextual interference schedule is deemed to produce a high cognitive effort reflecting the amount of cognitive processing needed to perform a skill (Patterson and Lee, 2008). To manipulate cognitive load and, as a consequence, action monitoring levels, we constrained participants to adjust less frequently (low interference) or more frequently (high interference) their attention focus (see Table 1). Shifting the focus of attention (external or internal) from task to task every minute is expected to determine a higher conscious monitoring associated with the higher cognitive effort than shifting the focus every 3 min. Drawing on previous research findings showing the benefits of conscious monitoring (e.g., Bortoli et al., 2012; Schücker et al., 2014; van Ginneken et al., 2017), we hypothesized to find same levels of performance across the four conditions stemming from the relationship between monitoring levels and the external/internal focus. Moreover, we expected the four conditions to result in better performance and running economy compared to a baseline and a follow up without attentional constraints. Together with performance and running economy, measured as time-to-exhaustion and rate of oxygen consumption, we assessed perceived exertion and core affect (i.e., arousal and hedonic tone). Higher levels of exertion during the endurance task were expected to be accompanied by higher levels of perceived exertion, and lower levels of perceived arousal (less energy) and hedonic tone (more displeasure). These predictions are consistent with previous research results. For instance, in a study with orienteering athletes Robazza et al. (2018) showed a similar pattern of results between perceived exertion and emotion-related (psychobiosocial) states dysfunctional for performance.

**Table 1:** Four Experimental Schedules derived from the interplay between Low/High conscious monitoring and External/Internal attention focus.

Attention focus	Conscious monitoring	
	High – Type 2 performance	Low – Type 1 performance
External	On a metronome for 1 min On a bellows for 1 min On a ball for 1 min	On a metronome for 3 min On a bellows for 3 min On a ball for 3 min
Internal	On feet rhythm for 1 min On breathing rhythm for 1 min On arms and shoulders rhythm for 1 min	On feet rhythm for 3 min On breathing rhythm for 3 min On arms and shoulders rhythm for 3 min

*The sequence in each schedule is repeated until exhaustion.*

## Materials and Methods

### Participants

Sample size was determined using G^∗^Power 3.1 (Faul et al., 2007). Consistent with previous studies using similar designs (Schücker et al., 2016a,b), we set the following input parameters for *a priori* power analysis: *f* = 0.25, α = 0.05, 1-β = 0.95, 1 group, 6 measurements, *r*_repeated measures_ = 0.50, ε = 1. The required sample size was 28 participants. To account for possible dropouts, we involved in the study 34 participants. Two of them discontinued participation from the study due to personal reasons. Therefore, 32 male participants, aged between 20 and 44 years (*M*_age_ = 29.12 years, *SD* = 6.12 years) were engaged in this investigation on a voluntary basis. All of them were healthy and free from injuries, engaged regularly in different physical activities of low to moderate intensity, and were accustomed to treadmill running. On the initial visit, baseline assessment, obtained by an incremental cardiopulmonary exercise test (CPET; American College of Sports Medicine, 2018), showed that the fitness level of participants was 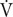
O_2_max *M* = 54.02 ml Kg^−1^ m^−1^, *SD* = 9.05, HRmax *M* = 184.41, *SD* = 10.88. Participants were informed about the procedure, the measurements, and the general purpose of the study, and provided written informed consent before participation. The study was conducted in accordance with the Declaration of Helsinki. Furthermore, the study was approved by the ethics committee of the University of Verona with anonymity, confidentiality, and allowance to leave the study at any point without any consequences being assured for the participants.

### Measures

#### Running Performance

The running performance during each time-to-exhaustion (TTE) test was measured as the time the participants reached volitional exhaustion.

#### Metabolic Measures

Participants completed a CPET to volitional exhaustion on a treadmill (RunRace, Technogym, Gambettola, Italy) with continuous breath-by-breath respiratory gas exchange measurement to record their oxygen consumption (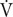
O_2_, ml/min/kg). 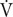
O_2_ was measured using an online metabolic cart calibrated before each test (Quark C-PET; Cosmed Srl, Rome, Italy). Performers were required to wear a breathing mask, which was fitted individually. Heart rate (HR, bpm) was measured continuously using a chest belt by wireless telemetry (Cosmed HR monitor) which transmitted the HR signal to the spiroergometric system.

#### Blood Measures

The lactate profile (mmol/L) of participants was measured by taking blood samples from the earlobe. The blood samples were examined with an enzymatic-amperometric blood lactate analyzer (Biosen C-Line; EKF Diagnostics, Cardiff, United Kingdom) according to the manufacturer’s instructions.

#### Ratings of Perceived Exertion (RPE)

The Borg CR-10 RPE scale (Borg, 1998) was administered to measure perceived exertion. Perceived exertion is proposed to complement physiological assessment in a wide range of areas, including exercise and sports. The Borg CR-10 RPE scale measures the perceived exertion on an interval using verbal anchors: 0 = *nothing at all*, 0.5 = *extremely weak*, 1 = *very weak*, 2 = *weak*, 3 = *moderate*, 5 = *strong*, 7 = *very strong*, 10 = *extremely strong*, • = *absolute maximum*. For 4, 6, 8, and 9 no verbal anchors are used. The score of 11 is assigned to *absolute maximum*.

#### Affect Grid

Russell et al. (1989) designed an affect grid to quickly assess core affect (i.e., arousal and hedonic tone) along the dimensions of sleepiness-energy and displeasure-pleasure. Following an explanation of the terms *arousal* and *hedonic tone*, the performer is asked to check a box within a 9 × 9 grid or to provide a verbal report that represents the perceived arousal and hedonic tone intensity at that moment. Hence, both the sleepiness-energy and the pleasure-displeasure dimensions could range from 1 to 9. Higher numbers indicate higher energy and higher pleasure levels, while lower numbers correspond to higher sleepiness and higher displeasure levels. This scale enables quick and low invasive assessment of one’s introspective states during performance.

#### Manipulation Check

After the TTE tests the participants were asked to rate on a 10-point scale, ranging from 0 = *never* to 10 = *always*, the frequency they were able to focus attention externally or internally following the instructions provided them according to the experimental manipulations. The questions were: “*How often did you pay attention to the rhythm of the metronome, bellows, and ball?*” (low/high monitoring and external focus conditions), “*How often did you pay attention to the rhythm of the feet, breathing, and arms and shoulders?*” (low/high monitoring and internal focus conditions).

### Procedure

The experimental protocol consisted of seven visits to the laboratory with intervisit intervals of 48 h. Collection of data occurred in an environmental controlled condition (temperature fixed at 20°C, no other people allowed in the laboratory, no music played). All trials were performed with the treadmill slope maintained at 1%.

#### Incremental Test

During the first visit the participants had the opportunity to familiarize with the experimental setting. They were instructed about the use of the Borg CR-10 RPE scale and the affect grid, and then completed a treadmill (RunRace, Technogym, Gambettola, Italy) cardiorespiratory incremental stress test. Breath-by-breath (Quark CPET, Cosmed, Rome, Italy) 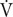
O_2_ and carbon dioxide production, ventilation, and heart rate data were collected. After a 4 min of 8 km h^−1^ warm-up, the velocity was incremented by 0.1 km h^−1^ each 6 s up to voluntary exhaustion. Treadmill inclination was kept fixed at 1% throughout the test. Maximal oxygen consumption (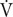
O_2_max) was obtained at the last 30 s of the test. Blood lactate concentration, from the earlobe, was measured at the third minute of recovery (Biosen C-Line, EKF Diagnostics, Cardiff, United Kingdom). In all tests, maximal heart rate was greater than 90% of age-predicted maximum value and respiratory quotient was greater than 1.10. The second ventilatory threshold (VT_2_), also described as respiratory compensation point, was identified blindly by two operators at the simultaneous increasing of both ventilatory equivalents for O_2_ (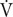
E/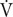
O_2_) and for CO_2_ (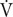
E/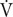
CO_2_; Wasserman et al., 1994). Oxygen consumption (ml kg^−1^ min^−1^), heart rate (bpm), and work velocity (km h^−1^) at VT_2_ were calculated.

#### Time-to-Exhaustion (TTE) Test at Individual Constant Load

During the second visit, participants performed a TTE test conducted without attentional manipulation conditions. In this baseline assessment, performers started with a 1-min resting period standing still on the treadmill, followed by a 4-min warm-up stage running at a constant individual speed calculated as the 50% of the individual 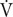
O_2_max. After 180 s run, the individual preferred running rate (PRR) was calculated as the number of steps on the treadmill in 1 min run at individual 105% of VT_2_ measured at steady state condition. Participants were then asked to run until exhaustion at their constant individual speed calculated as the 105% of VT_2_. During the following four visits to the laboratory, in which the attention focus was manipulated, the participants completed TTE tests running until exhaustion at a constant individual speed corresponding to the 105% of VT_2_. The protocol of each TTE test was the same described for the second visit. TTE was considered as the maximum interval (sec) in which the participants could maintain the running intensity assigned until volitional exhaustion. During each test, individual PRR was set and controlled for each participant.

Participants were assigned in a counterbalanced order to one of the four attentional conditions each one occurring on a different day. The four conditions resulted from the interaction between conscious monitoring (low and high) and attention focus (external and internal; see Table 1). Consistent with the MAP model predictions (Bortoli et al., 2012; Robazza et al., 2016), low or high monitoring levels (either external or internal) were hypothesized to be associated with a Type 1 or a Type 2 performance state, respectively. In the external focus condition, a laptop and a slide projector were positioned in front of the treadmill to clearly project on a white wall three videos of a metronome, a bellows, and a ball. The projected videos were visible at 1.5 m from the participants’ eye height. An amplifier and two audio speakers guaranteed a clear perception of the sound associated with the metronome, bellows, and ball moving rhythmically in synchrony with the individual PRR rhythm. Participants were requested to focus attention during running on the videos and the related sound. The projection of the metronome, bellows, and ball was alternated every 3 min or 1 min in the low or high monitoring conditions, respectively, until exhaustion. In the internal focus condition, participants were instructed to pay close attention to the rhythmic movement of feet, breathing, and arms and shoulders associated with running. Similar to the external focus condition, the internal focus on the rhythm of the different bodily parts were alternated every 3 min or 1 min until exhaustion. Reminders to pay close attention to the videos or to the physical sensations were systematically provided to performers. A follow up TTE test without manipulation of the attentional focus was conducted in a final visit to the laboratory 48 hr later.

Ratings of perceived exertion and affective states (arousal and hedonic tone) data were collected in the last 5 s of the 1-min resting period, in the last 5 s of the first minute of each TTE test, and in the last 5 s of every 3 min period throughout the entire test. During the last 30 s after the end of each TTE test, blood samples were taken from the earlobe of the participants to measure the blood lactate concentration. We collected breath-by-breath (Quark CPET; Cosmed, Rome, Italy) and calculated the mean steady state value during the last 30 s of the test for 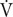
O_2_, ventilation, and heart rate. Steady state blood lactate concentration, from the earlobe, was measured at the end of each trial (Biosen C-Line, EKF Diagnostics, Cardiff, United Kingdom). After a 4 min of 8 km h^−1^ warm-up, the running speed was maintained at 105% of second VT_2_ velocity, with an inclination fixed at 1% until voluntary exhaustion. Steady state 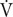
O_2_ was calculated as the mean during the last 30 s of the test.

### Data Analysis

Repeated-measures analysis of variance (RM-ANOVA) was performed to examine the effect of the experimental conditions (i.e., baseline, four experimental conditions, and follow up) on the study variables (i.e., performance, 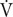
O_2_, blood lactate levels, RPE, and perceived arousal and hedonic tone) across six visits to the laboratory and six data collections of each variable within a session. The six data values of each variable entered in the analysis corresponded to the 1-min resting period (baseline), isotimes 0% (first full minute), 25, 50, 75, and 100% (last completed minute) in the TTE test. The isotime at 100% was defined as the shortest TTE time attained by a performer in the four tests. The minute identified as 100% isotime was multiplied by 0.25, 0.5, and 0.75 to establish 25, 50, and 75% isotime values, respectively (see Blanchfield et al., 2014). RM-ANOVA was also conducted on the manipulation check data.

## Results

Descriptive statistics of study variables are reported in Tables 2, 3. In the RM-ANOVAs, the assumption of sphericity was violated, and thus the Greenhouse-Geisser correction was applied to the degrees of freedom for *F* statistic calculation. As can be seen from the results in Table 4, the only significant differences at *p* < 0.01 were found across the assessment phases (i.e., data collection within a session) for RPE, perceived arousal, and hedonic tone. The increasing levels of exertion through the task were accompanied by higher RPE, as well as lower ratings of perceived arousal (less energy) and hedonic tone (more displeasure), which indicate a worsening of the individual mental state during the TTE test. Significant differences were not observed either on experimental conditions or on the interaction between experimental conditions and assessment. Of note, manipulation check results indicate that performers were able to adhere satisfactorily to the experimental conditions given that mean ratings were between 7 and 8 (i.e., *much* and *very much*) on the 10-point scale.

**Table 2:** Descriptive Statistics of Study Variables across Experimental Schedules.

Variable	Schedule	*M* ± *SD*	% Change from baseline
Manipulation check	Low monitoring, external focus	7.81 ± 1.26	
	Low monitoring, internal focus	7.64 ± 1.07	
	High monitoring, external focus	8.09 ± 1.00	
	High monitoring, internal focus	7.41 ± 1.06	
Lactate (mmol/L)	Baseline	9.31 ± 3.07	
	Low monitoring, external focus	8.78 ± 2.60	−5.70
	Low monitoring, internal focus	9.16 ± 2.33	−1.68
	High monitoring, external focus	9.13 ± 2.37	−2.01
	High monitoring, internal focus	8.84 ± 2.44	−5.03
	Follow-up	8.50 ± 2.20	−8.72
Performance (sec)	Baseline	335.12 ± 147.43	
	Low monitoring, external focus	350.16 ± 150.71	4.49
	Low monitoring, internal focus	337.44 ± 126.54	0.69
	High monitoring, external focus	352.66 ± 120.19	5.23
	High monitoring, internal focus	356.19 ± 144.93	6.28
	Follow-up	345.00 ± 127.32	2.95

**Table 3:** Descriptive Statistics (M ± SD) of Study Variables across Experimental Schedules and Six Data Collections.

Variable	Schedule	Baseline	Isotime				
			0%	25%	50%	75%	100%
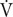 O_2_ (ml/min)	Baseline	453 ± 96	2478 ± 368	3478 ± 673	3800 ± 669	3935 ± 684	3944 ± 679
	Low, external	432 ± 115	2470 ± 340	3454 ± 614	3833 ± 657	3980 ± 680	4036 ± 657
	Low, internal	462 ± 112	2439 ± 323	3428 ± 616	3777 ± 646	3938 ± 664	3949 ± 649
	High, external	435 ± 126	2488 ± 400	3522 ± 629	3835 ± 637	3989 ± 658	4025 ± 650
	High, internal	456 ± 167	2455 ± 362	3472 ± 654	3829 ± 643	3978 ± 670	4011 ± 656
	Follow-up	467 ± 93	2446 ± 372	3479 ± 601	3837 ± 588	3977 ± 597	4038 ± 559
RPE	Baseline	1.38 ± 0.98	3.06 ± 0.86	4.78 ± 1.18	6.24 ± 1.50	8.58 ± 1.88	8.70 ± 1.49
	Low, external	1.25 ± 0.68	3.14 ± 1.00	4.84 ± 1.46	6.44 ± 1.47	8.52 ± 1.44	8.42 ± 1.44
	Low, internal	1.27 ± 0.78	3.26 ± 1.34	5.44 ± 3.47	6.54 ± 1.44	8.52 ± 1.56	8.58 ± 0.95
	High, external	1.28 ± 0.78	2.99 ± 0.97	4.68 ± 1.35	6.32 ± 1.88	8.55 ± 1.28	8.55 ± 1.25
	High, internal	1.14 ± 0.71	2.73 ± 0.89	4.37 ± 1.33	6.10 ± 1.66	8.27 ± 1.96	8.70 ± 1.64
	Follow-up	1.22 ± 0.76	2.90 ± 0.95	4.58 ± 1.27	6.31 ± 1.18	8.60 ± 1.25	8.68 ± 1.28
Sleepiness-energy (arousal)	Baseline	6.50 ± 1.52	5.83 ± 1.17	5.22 ± 1.26	4.60 ± 1.59	3.66 ± 2.13	3.66 ± 2.13
	Low, external	6.44 ± 1.34	5.84 ± 1.12	5.29 ± 1.24	4.55 ± 1.43	3.38 ± 1.84	4.13 ± 2.15
	Low, internal	6.44 ± 1.19	6.01 ± 1.20	5.43 ± 1.25	4.82 ± 1.61	3.69 ± 1.51	3.69 ± 1.91
	High, external	6.69 ± 1.12	6.15 ± 1.03	5.57 ± 1.27	4.99 ± 1.52	4.00 ± 2.00	3.66 ± 1.64
	High, internal	6.69 ± 1.38	6.20 ± 1.07	5.66 ± 1.04	4.91 ± 1.44	3.91 ± 2.01	3.59 ± 1.62
	Follow-up	6.41 ± 1.19	5.99 ± 1.03	5.52 ± 1.07	4.81 ± 1.31	3.66 ± 1.79	3.69 ± 1.77
Displeasure-pleasure (hedonic tone)	Baseline	5.47 ± 1.54	4.92 ± 1.49	4.26 ± 1.68	3.83 ± 1.64	2.78 ± 1.62	2.78 ± 1.62
	Low, external	5.88 ± 1.93	5.04 ± 1.85	4.55 ± 1.99	3.98 ± 1.97	3.00 ± 1.61	3.00 ± 1.85
	Low, internal	5.56 ± 1.68	5.04 ± 1.71	4.67 ± 3.05	3.72 ± 1.95	2.72 ± 1.49	2.78 ± 1.29
	High, external	5.56 ± 1.72	4.97 ± 1.70	4.42 ± 1.81	3.96 ± 1.79	3.06 ± 1.70	3.41 ± 1.85
	High, internal	6.00 ± 1.41	5.37 ± 1.37	4.55 ± 1.52	3.84 ± 1.61	3.59 ± 2.17	2.78 ± 1.54
	Follow-up	5.76 ± 1.50	5.44 ± 1.42	5.03 ± 1.62	4.61 ± 1.76	3.63 ± 1.91	3.66 ± 1.93

**Table 4:** Analysis of Variance Results across Experimental Schedules and Six Data Collections (Assessment).

Variables Effects	*F*(df)	*p*	η_p_^2^	Power
Manipulation check				
Experimental schedules	3.472 (2.618, 81.159)	0.025	0.101	0.716
Lactate				
Experimental schedules	1.102 (3.872, 120.017)	0.358	0.034	0.332
Performance				
Experimental schedules	0.377 (2.687, 83.307)	0.748	0.012	0.118
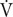 0_2_max				
Experimental schedules	1.368 (4.286, 132.861)	0.246	0.042	0.433
Assessment	839.287 (1.238, 38.388)	<0.001	0.964	1.000
Experimental × assessment	1.158 (10.600, 328.609)	0.101	0.049	0.796
RPE				
Experimental schedules	0.918 (1.518, 44.016)	0.386	0.031	0.178
Assessment	227.224 (1.575, 45.669)	<0.001	0.887	1.000
Experimental × assessment	1.196 (1.216, 35.271)	0.291	0.038	0.195
Sleepiness-energy (arousal)				
Experimental schedules	0.982 (3.719, 115.298)	0.416	0.031	0.292
Assessment	48.137 (1.157, 35.863)	<0.001	0.608	1.000
Experimental × assessment	1.375 (7.851, 243.392)	0.209	0.042	0.615
Displeasure-pleasure (hedonic tone)				
Experimental schedules	2.324 (3.903, 120.998)	0.062	0.070	0.652
Assessment	92.649 (1.566, 48.559)	<0.001	0.749	1.000
Experimental × assessment	1.236 (8.006, 248.178)	0.278	0.038	0.566

*Greenhouse-Geisser correction was applied to the degrees of freedom (df); η_p_^2^ = partial eta squared.*

## Discussion

The purpose of the study was to establish whether low or high levels of external or internal conscious monitoring would influence performance in an endurance task. As expected, the four experimental conditions derived from the interplay between low/high conscious monitoring and external/internal attention focus did not yield significant results in the study variables (i.e., 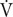
O_2_, blood lactate levels, RPE, and perceived arousal and hedonic tone). Same performance levels (i.e., TTE) were also observed irrespective of whether participants used a high or low level of action monitoring through an external or internal focus of attention. Contrary to what was expected, the experimental conditions did not determine better performance compared to baseline and follow up. Also, significant results did not emerge across sessions in the other study variables. The lack of significant differences was probably due to the good physical activity level of participants who regularly engaged in endurance tasks, including running and cycling, during their leisure time. Experienced participants, acquainted with conditions of strain and fatigue and highly motivated toward endurance tasks, had most likely developed personal strategies to deal with endurance requirements.

Attentional focus in endurance sports has been found to largely affect performance (e.g., Brick et al., 2016a; McCormick et al., 2018; Robazza et al., 2018). Dealing effectively with task demands, physical exertion, and mental strain in high achievement, pressurized contexts, involves one’s monitoring of both internal (e.g., bodily) sensations and external (e.g., environmental) stimuli (Carson and Collins, 2016; Gropel, 2016; Buchanan et al., 2018) to actively regulate the own pacing and strategy (Brick et al., 2014). Internal sensory monitoring (e.g., breathing, muscles soreness, fatigue), outward monitoring (e.g., other competitors, split times, route, mile markers, water stations), and active self-regulation (e.g., cadence, pacing, technique, strategy, muscle relaxation) are also emphasized in Brick et al. (2014) working model of attentional focus in endurance activity. Thus, results of the current study offer further support to the use of both internal and external attentional foci in endurance tasks. Notably, the study design required performers to monitor internal or external information while executing rather than directing attention to the control of automated processes. This design is in accordance with Schücker et al. (2014) classification of the attention focus on physical sensations helpful for performance, and a focus on automated processes hampering performance. Results can also be interpreted in light of the reinvestment theory (Masters, 1992; Masters and Maxwell, 2008) and explicit monitoring theory (Beilock and Carr, 2001; Beilock, 2011), which contend that directing attention on movement execution hinders automaticity. In these views, detrimental effects are contended to derive from a voluntary control of movement automatisms. On the other hand, beneficial effects of an internal focus are supposed to be in function of conscious monitoring, which involves awareness of the course of action without necessarily changing it (van Ginneken et al., 2017).

Results of our study can also be understood within the MAP model conceptualization (Bortoli et al., 2012; Robazza et al., 2016) inasmuch as different levels of conscious monitoring (low or high) of the core components of the action typify Type 1 and Type 2 functional states for performance. In particular, a low level of conscious monitoring characterizes a Type 1 state, while a high level of monitoring is a facet of a Type 2 state. Type 3 and Type 4 dysfunctional states, not examined in this study, are assumed to either involve excessive attention toward the execution of an automated action (Type 3) or wandering attention (Type 4). It is therefore critical for optimal performance to identify the core components of the action, especially when executing demanding tasks or strenuous activities, dealing with novel problems, fatigue, and unexpected events, or performing under competitive pressure. Under these circumstances, reinvesting attention in the task can be inevitable (Masters and Maxwell, 2008) and beneficial, as long as the athlete is able to direct attention on previously identified core components of the action (Hanin and Hanina, 2009; Hanin et al., 2016).

According to the MAP perspective (Bortoli et al., 2012; Robazza et al., 2016), core components are not those highly automated elements of the technique that are consistently executed in different conditions without conscious attentional control. Core components are instead conceived as fundamental actions or action-related behaviors that fluctuate in accuracy especially under challenging situations. Examples of core components are “grip” and “aiming” in shooting sports (Bortoli et al., 2012; Robazza et al., 2016), “elbow alignment” and “attention on target” in dart-throwing (Bertollo et al., 2013), “acceleration after the curve” and “braking modulation” in racecar driving (Filho et al., 2015). In a TTE, endurance cycling activity (Bertollo et al., 2015; di Fronso et al., 2018), external pacing focusing attention on metronome beats and internal pacing directing attention to feet rhythm while pedaling represented two core components of the task associated with Type 1 and Type 2 performance, respectively. In our study alike, we used external pacing (i.e., metronome, bellows, and ball moving rhythm) and internal pacing (i.e., feet, breathing, and arms/shoulders rhythm) as core components of the endurance task to attain Type 1 and Type 2 optimal performance states. However, previous studies did not establish whether different levels of external or internal monitoring of the core components of the action influence performance differently. Findings of the current study suggest that both external and internal foci of attention to specific action elements of a treadmill endurance task, which can be considered core components, have same effect on performance, regardless of the low or high levels of monitoring.

The MAP model conceptualization of different states associated with optimal performance is akin to other theoretical views (for a review, see Robazza and Ruiz, 2018). For example, the default-interventionist framework (Evans and Stanovich, 2013), within the dual-process theories, maintains that human behavior is governed by both automatic and controlled modes of processing (see Furley et al., 2015; Furley and Wood, 2016). Accordingly, Type 1 (default) autonomous processing is “intuitive,” does not rely on working memory and controlled attention, and enables fast and effortless behavioral responses initiated in the presence of relevant triggering conditions. In contrast, Type 2 (interventionist) controlled processing relies on working memory capacity, involves intentional behavior, and is appropriate when facing novel problems or new challenges. Expert performers can move between the two types of processing to cope with pressure and adapt to the competitive demands. Toner et al. (2016) expressed similar positions while discussing the pre-reflective and reflective modes of functioning in the Colombetti (2011) taxonomy of bodily self-awareness. Toner et al. argued that “some performers may be perfectly used to *monitoring* and *controlling* certain aspects of their movement in order to maintain performance proficiency.” (p. 308, italic added). Swann et al. (2016, 2017a,b) also proposed that superior performance can be reached both in a flow state (a condition of effortless attention and automatic experience) and a clutch state attitude (an effortful condition with deliberate focus on the task), and that athletes can alternate from one state to the other during performance. The MAP model conceptualization also fits with the attentional control theory (Eysenck et al., 2007; Eysenck and Wilson, 2016), which distinguishes performance effectiveness (i.e., quality of performance) from processing efficiency (i.e., resources expended in the achievement process). Framed within the MAP model, performance effectiveness typifies Type 2 effortful performance (top-down control), while processing efficiency underlies Type 1 effortless performance (bottom-up control; Bertollo et al., 2016).

## Conclusion

Findings of our study have both theoretical and practical implications. Construed within the MAP model and other theoretical approaches, the results support the notion that internal and external monitoring in endurance sports can be equally effective and important for athletes to self-regulate pacing and deal with mental and physical strain (Brick et al., 2014). Our findings suggest this claim holds true regardless the levels of voluntary monitoring are low or high. Athletes should identify their most effective inward (e.g., breathing rhythm) and outward (e.g., mile markers) attentional foci, and be able to alternate between them to adjust pacing in function of their mental state, physical condition, and current situation. Switching among different low/high and internal/external monitoring strategies might complement other associative and dissociative psychological strategies (e.g., goal setting, imagery, self-talk; McCormick et al., 2015, 2018) aimed to manage inner states and situational demands.

Some limitations of the study should be acknowledged for future research. Individual strategies to deal with the endurance task were not examined prior to the study. These may explain the lack of performance improvements across the intervention in comparison to the baseline. Investigating already developed personal strategies and preferences can provide information on how to develop applied interventions to suit individual needs. Moreover, the focus of the study was on functional task monitoring. To avoid excessive burden on the participants, dysfunctional attention toward the control of action or unrelated tasks was not included. Future studies may consider manipulating attention to ensure that participants experience all four performance states as conceptualized in the MAP model (Bortoli et al., 2012; Robazza et al., 2016) or related multiple states (MuSt; Robazza and Ruiz, 2018) perspectives. Finally, other limitations are that the investigation was conducted in the laboratory and that the cognitive effort induced using the contextual interference paradigm may not have been high. More ecologically valid studies can establish what kind of monitoring strategies are more effective and transferable to the field.

## Data Availability

The datasets generated for this study are available on request to the corresponding author.

## Author Contributions

All authors listed have made a substantial, direct and intellectual contribution to the work, and approved it for publication.

## Conflict of Interest Statement

The authors declare that the research was conducted in the absence of any commercial or financial relationships that could be construed as a potential conflict of interest.

## References

[B1] American College of Sports Medicine (2018). *ACSACSM’s Guidelines for Exercise Testing and Prescription, 10th EdnM’s Guidelines for Exercise Testing and Prescription*, 10th Edn. Philadelphia, PA: Wolters Kluwer Health.

[B2] Beilock S. L. (2011). *Choke. The Secret to Performing Under Pressure.* London: Constable & Robinson.

[B3] Beilock S. L. Carr T. H. (2001). On the fragility of skilled performance: what governs choking under pressure? *J. Exp. Psychol. Gen.* 130 701–725. 10.1037/0096-3445.130.4.701 11757876

[B4] Bertollo M. Bortoli L. Gramaccioni G. Hanin Y. Comani S. Robazza C. (2013). Behavioural and psychophysiological correlates of athletic performance: a test of the multi-action plan model. *Appl. Psychophysiol. Biofeedback* 38 91–99. 10.1007/s10484-013-9211-z 23483293

[B5] Bertollo M. di Fronso S. Filho E. Conforto S. Schmid M. Bortoli L. (2016). Proficient brain for optimal performance: the MAP model perspective. *PeerJ* 4:e2082. 10.7717/peerj.2082 27257557 PMC4888308

[B6] Bertollo M. di Fronso S. Filho E. Lamberti V. Ripari P. Reis V. M. (2015). To focus or not to focus: is attention on the core components of action beneficial for cycling performance? *Sport Psychol.* 29 110–119. 10.1123/tsp.2014-0046

[B7] Blanchfield A. W. Hardy J. Marcora S. M. (2014). Non-conscious visual cues related to affect and action alter perception of effort and endurance performance. *Front. Hum. Neurosci.* 8:967. 10.3389/fnhum.2014.00967 25566014 PMC4263011

[B8] Boccia G. Dardanello D. Tarperi C. Festa L. La Torre A. Pellegrini B. (2018). Women show similar central and peripheral fatigue to men after half-marathon. *Eur. J. Sport Sci.* 8 695–704. 10.1080/17461391.2018.1442500 29490592

[B9] Borg G. (1998). *Borg’s Perceived Exertion and Pain Scales.* Champaign, IL: Human Kinetics.

[B10] Bortoli L. Bertollo M. Hanin Y. Robazza C. (2012). Striving for excellence: a multi-action plan intervention model for shooters. *Psychol. Sport Exerc.* 13 693–701. 10.1016/j.psychsport.2012.04.006

[B11] Brick N. MacIntyre T. Campbell M. (2014). Attentional focus in endurance activity: new paradigms and future directions. *Int. Rev. Sport Exerc. Psychol.* 7 106–134. 10.1080/1750984X.2014.885554

[B12] Brick N. E. Campbell M. J. Metcalfe R. S. Mair J. L. Macintyre T. E. (2016a). Altering pace control and pace regulation: attentional focus effects during running. *Med. Sci. Sports Exerc.* 48 879–886. 10.1249/mss.0000000000000843 26673128

[B13] Brick N. E. MacIntyre T. E. Campbell M. J. (2016b). Thinking and action: a cognitive perspective on self-regulation during endurance performance. *Front. Physiol.* 7:159. 10.3389/fphys.2016.00159 27199774 PMC4847621

[B14] Buchanan J. J. Park I. Chen J. Mehta R. K. McCulloch A. Rhee J. (2018). Expert monitoring and verbal feedback as sources of performance pressure. *Acta Psychol.* 186 39–46. 10.1016/j.actpsy.2018.04.009 29698846

[B15] Carson H. J. Collins D. (2016). The fourth dimension: a motoric perspective on the anxiety–performance relationship. *Int. Rev. Sport Exerc. Psychol.* 9 1–21. 10.1080/1750984X.2015.1072231 26692896 PMC4662095

[B16] Colombetti G. (2011). Varieties of pre-reflective self-awareness: foreground and background bodily feelings in emotion experience. *Inquiry* 54 293–313. 10.1080/0020174X.2011.575003

[B17] di Fronso S. Tamburro G. Robazza C. Bortoli L. Comani S. Bertollo M. (2018). Focusing attention on muscle exertion increases EEG coherence in an endurance cycling task. *Front. Psychol.* 9:1249. 10.3389/fpsyg.2018.01249 30079045 PMC6063037

[B18] Evans J. S. B. T. Stanovich K. E. (2013). Dual-process theories of higher cognition: advancing the debate. *Perspect. Psychol. Sci.* 8 223–241. 10.1177/1745691612460685 26172965

[B19] Eysenck M. W. Derakshan N. Santos R. Calvo M. G. (2007). Anxiety and cognitive performance: attentional control theory. *Emotion* 7 336–353. 10.1037/1528-3542.7.2.336 17516812

[B20] Eysenck M. W. Wilson M. R. (2016). “Sporting performance, pressure and cognition-Introducing attentional control theory: sport,” in *An Introduction to Applied Cognitive Psychology*, 2nd Edn, eds Groome D. Eysenck M. W. (New York, NY: Routledge), 329–350.

[B21] Farrow D. Buszard T. (2017). “Exploring the applicability of the contextual interference effect in sports practice,” in *Sport and the Brain: The Science of Preparing, Enduring and Winning (Part B)*, eds Wilson M. R. Walsh V. Parkin B. (Cambridge, MA: Academic Press), 69–83. 10.1016/bs.pbr.2017.07.002 29031473

[B22] Faul F. Erdfelder E. Lang A.-G. Buchner A. (2007). G^∗^Power 3: a flexible statistical power analysis program for the social, behavioral, and biomedical sciences. *Behav. Res. Methods* 39 175–191. 10.3758/BF03193146 17695343

[B23] Filho E. Di Fronso S. Mazzoni C. Robazza C. Bortoli L. Bertollo M. (2015). My heart is racing! Psychophysiological dynamics of skilled racecar drivers. *J. Sports Sci.* 33 945–959. 10.1080/02640414.2014.977940 25555177

[B24] Furley P. Schweizer G. Bertrams A. (2015). The two modes of an athlete: dual-process theories in the field of sport. *Int. Rev. Sport Exerc. Psychol.* 8 106–124. 10.1080/1750984X.2015.1022203

[B25] Furley P. Wood G. (2016). Working memory, attentional control, and expertise in sports: a review of current literature and directions for future research. *J. Appl. Res. Mem. Cogn.* 5 415–425. 10.1016/j.jarmac.2016.05.001

[B26] Gardner F. L. Moore Z. E. (2007). *The Psychology of Enhancing Human Performance: The Mindfulness-Acceptance-Commitment (MAC) Approach.* New York, NY: Springer 10.1891/9780826103369

[B27] Gropel P. (2016). Self-focused attention and motor skill failure: the moderating role of action orientation. *Sport Exerc. Perform. Psychol.* 5 206–217. 10.1037/spy0000059

[B28] Hanin Y. Hanina M. (2009). Optimization of performance in top-level athletes: an action-focused coping approach. *Int. J. Sports Sci. Coach.* 4 47–58. 10.1260/1747-9541.4.1.47

[B29] Hanin Y. Hanina M. Šašek H. Kobilšek A. (2016). Emotion-centered and action-centered coping in elite sport: task execution design approach. *Int. J. Sports Sci. Coach.* 11 566–588. 10.1177/1747954116654782

[B30] Lohse K. Sherwood D. (2011). Defining the focus of attention: effects of attention on perceived exertion and fatigue. *Front. Psychol.* 2:332. 10.3389/fpsyg.2011.00332 22102843 PMC3214735

[B31] Malhotra N. Poolton J. M. Wilson M. R. Uiga L. Masters R. S. W. (2015). Examining movement-specific reinvestment and performance in demanding contexts. *J. Sport Exerc. Psychol.* 37 327–338. 10.1123/jsep.2014-0220 26265344

[B32] Marcora S. M. Staiano W. (2010). The limit to exercise tolerance in humans: mind over muscle? *Eur. J. Appl. Physiol.* 109 763–770. 10.1007/s00421-010-1418-6 20221773

[B33] Marcora S. M. Staiano W. Manning V. (2009). Mental fatigue impairs physical performance in humans. *J. Appl. Physiol.* 106 857–864. 10.1152/japplphysiol.91324.2008 19131473

[B34] Martin K. Meeusen R. Thompson K. G. Keegan R. Rattray B. (2018). Mental fatigue impairs endurance performance: a physiological explanation. *Sports Med.* 48 2041–2051. 10.1007/s40279-018-0946-9 29923147

[B35] Masters R. Maxwell J. (2008). The theory of reinvestment. *Int. Rev. Sport Exerc. Psychol.* 1 160–183. 10.1080/17509840802287218

[B36] Masters R. S. W. (1992). Knowledge, knerves and know-how: the role of explicit versus implicit knowledge in the breakdown of a complex motor skill under pressure. *Br. J. Psychol.* 83 343–358. 10.1111/j.2044-8295.1992.tb02446.x 17470398

[B37] McCormick A. Meijen C. Anstiss P. A. Jones H. S. (2018). Self-regulation in endurance sports: theory, research, and practice. *Int. Rev. Sport Exerc. Psychol.* 10.1080/1750984X.2018.1469161 [Epub ahead of print].

[B38] McCormick A. Meijen C. Marcora S. (2015). Psychological determinants of whole-body endurance performance. *Sports Med.* 45 997–1015. 10.1007/s40279-015-0319-6 25771784 PMC4473096

[B39] McMorris T. Barwood M. Corbett J. (2018). Central fatigue theory and endurance exercise: toward an interoceptive model. *Neurosci. Biobehav. Rev.* 93 93–107. 10.1016/j.neubiorev.2018.03.024 29608992

[B40] Morgan W. P. Pollock M. L. (1977). Psychologic characterization of the elite distance runner. *Ann. N. Y. Acad. Sci.* 301 382–403. 10.1111/j.1749-6632.1977.tb38215.x 270929

[B41] Patterson J. T. Lee T. D. (2008). “Organizing practice: the interaction of repetition and cognitive effort for skilled performance,” in *Developing Sport Expertise: Researchers and Coaches put Theory into Practice*, eds Farrow D. Baker J. MacMahon C. (New York, NY: Routledge), 119–134.

[B42] Pedrinolla A. Li Volti G. Galvano F. Schena F. Perciavalle V. Di Corrado D. (2018). Bioenergetics and psychological profile of an ultra endurance walker. *J. Sports Med. Phys. Fit.* 58 549–554. 10.23736/S0022-4707.17.07088-8 28229566

[B43] Robazza C. Bertollo M. Filho E. Hanin Y. Bortoli L. (2016). Perceived control and hedonic tone dynamics during performance in elite shooters. *Res. Q. Exerc. Sport* 87 284–294. 10.1080/02701367.2016.1185081 27253427

[B44] Robazza C. Izzicupo P. D’Amico M. A. Ghinassi B. Crippa M. C. Di Cecco V. (2018). Psychophysiological responses of junior orienteers under competitive pressure. *PLoS One* 13:e0196273. 10.1371/journal.pone.0196273 29698498 PMC5919653

[B45] Robazza C. Ruiz M. C. (2018). Emotional self-regulation in sport and performance. *Oxford Res. Encycl. Psychol.* 10.1093/acrefore/9780190236557.013.154 [Epub ahead of print].

[B46] Russell J. A. Weiss A. Mendelsohn G. A. (1989). Affect grid: a single-item scale of pleasure and arousal. *J. Pers. Soc. Psychol.* 57 493–502. 10.1037/0022-3514.57.3.493

[B47] Schücker L. Fleddermann M. de Lussanet M. Elischer J. Böhmer C. Zentgraf K. (2016a). Focusing attention on circular pedaling reduces movement economy in cycling. *Psychol. Sport Exerc.* 27 9–17. 10.1016/j.psychsport.2016.07.002

[B48] Schücker L. Schmeing L. Hagemann N. (2016b). Look around while running! Attentional focus effects in inexperienced runners. *Psychol. Sport Exerc.* 27 205–212. 10.1016/j.psychsport.2016.08.013

[B49] Schücker L. Knopf C. Strauss B. Hagemann N. (2014). An internal focus of attention is not always as bad as its reputation: how specific aspects of internally focused attention do not hinder running efficiency. *J. Sport Exerc. Psychol.* 36 233–243. 10.1123/jsep.2013-0200 24918307

[B50] Smits B. L. M. Pepping G. J. Hettinga F. J. (2014). Pacing and decision making in sport and exercise: the roles of perception and action in the regulation of exercise intensity. *Sports Med.* 44 763–775. 10.1007/s40279-014-0163-0 24706362

[B51] Stevinson C. D. Biddle S. J. H. (1998). Cognitive orientations in marathon running and “hitting the wall”. *Br. J. Sports Med.* 32 229–234. 10.1136/bjsm.32.3.229 9773172 PMC1756097

[B52] Stevinson C. D. Biddle S. J. H. (1999). Cognitive strategies in running: a response to Masters and Ogles (1998). *Sport Psychol.* 13 235–236. 10.1123/tsp.13.2.235

[B53] Swann C. Crust L. Jackman P. Vella S. A. Allen M. S. Keegan R. (2017a). Psychological states underlying excellent performance in sport: toward an integrated model of flow and clutch states. *J. Appl. Sport Psychol.* 29 375–401. 10.1080/10413200.2016.1272650

[B54] Swann C. Crust L. Vella S. A. (2017b). New directions in the psychology of optimal performance in sport: flow and clutch states. *Curr. Opin. Psychol.* 16 48–53. 10.1016/j.copsyc.2017.03.032 28813354

[B55] Swann C. Keegan R. Crust L. Piggott D. (2016). Psychological states underlying excellent performance in professional golfers: “Letting it happen” vs. “making it happen”. *Psychol. Sport Exerc.* 23 101–113. 10.1016/j.psychsport.2015.10.008

[B56] Toner J. Montero B. G. Moran A. (2016). Reflective and prereflective bodily awareness in skilled action. *Psychol. Conscious.* 3 303–315. 10.1037/cns0000090

[B57] van Ginneken W. F. Poolton J. M. Masters R. S. W. Capio C. M. Kal E. C. van der Kamp J. (2017). Comparing the effects of conscious monitoring and conscious control on motor performance. *Psychol. Sport Exerc.* 30 145–152. 10.1016/j.psychsport.2017.03.001 11432160

[B58] Wasserman K. Stringer W. W. Casaburi R. Koike A. Cooper C. B. (1994). Determination of the anaerobic threshold by gas-exchange: biochemical considerations, methodology and physiological-effects. *Z. Kardiol.* 83(Suppl. 3), 1–12. 7941654

[B59] Wulf G. (2007). Attentional focus and motor learning: a review of 10 years of research. *Bewegung Train.* 1 4–14.

[B60] Wulf G. (2013). Attentional focus and motor learning: a review of 15 years. *Int. Rev. Sport Exerc. Psychol.* 6 77–104. 10.1080/1750984x.2012.723728

[B61] Wulf G. Lewthwaite R. (2016). Optimizing performance through intrinsic motivation and attention for learning: the OPTIMAL theory of motor learning. *Psychon. Bull. Rev.* 23 1382–1414. 10.3758/s13423-015-0999-9 26833314

[B62] Wulf G. McNevin N. Shea C. H. (2001). The automaticity of complex motor skill learning as a function of attentional focus. *Q. J. Exp. Psychol. A* 54 1143–1154. 10.1080/02724980143000118 11765737

[B63] Zhang C.-Q. Si G. Duan Y. Lyu Y. Keatley D. A. Chan D. K. C. (2016). The effects of mindfulness training on beginners’ skill acquisition in dart throwing: a randomized controlled trial. *Psychol. Sport Exerc.* 22 279–285. 10.1016/j.psychsport.2015.09.005 21679770

